# Mood and suicidality amongst cyberbullied adolescents- a cross-sectional study from youth risk behavior survey

**DOI:** 10.1192/j.eurpsy.2021.255

**Published:** 2021-08-13

**Authors:** Y.C. Hsieh, P. Jain, N. Veluri, J. Bhela, B. Sheikh, F. Bangash, J. Gude, R. Subhedar, M. Zhang, M. Shah, Z. Mansuri, K. Aedma, T. Parikh

**Affiliations:** 1 School Of Public Health, Icahn School of Medicine at Mount Sinai, New York City, United States of America; 2 Psychiatry, State University of New York Upstate, Syracuse, United States of America; 3 N/a, American University of Integrative Sciences, School of Medicine, St.Michale, Barbados; 4 Psychiatry, Case Western Reserve / Metrohealth hospital, Cleveland, United States of America; 5 Psychiatry, Brookdale Hospital Medical Center, Brooklyn, United States of America; 6 Psychiatry, CJW Medical Center Richmond, Richmond, United States of America; 7 Psychiatry, Northwell Health/Loong Island Jewish Hospital, Queens, United States of America; 8 Psychiatry Department, McMaster University, Brampton, Canada; 9 Neuropsychiatry And Psychology, Johns Hopkins University, Baltimore, United States of America; 10 Psychiatry, Wright Center for Graduate Medical Education, Scranton, United States of America; 11 Department Of Psychiatry, Boston Children’s Hospital/Harvard Medical School, Boston, United States of America; 12 Psychiatry, Unitypoint Health, Peoria, United States of America; 13 Psychiatry, Ann & Robert H Lurie Children’s Hospital of Chicago, Chicago, United States of America

**Keywords:** Suicide, Depression, Youth Risk Behavior Survey, Cyberbully

## Abstract

**Introduction:**

There is a limited literature available showing mental health burden among adolescents following cyberbullying.

**Objectives:**

Aim is to evaluate the association of low mood and suicidality amongst cyberbullied adolescents.

**Methods:**

A study on CDC National Youth Risk Behavior Surveillance (YRBS) (1991-2017). Responses from adolescence related to cyberbullying and suicidality were evaluated. Chi-square and mix-effect multivariable logistic regression analysis was performed to find out the association of cyberbullying with sadness/hopelessness, suicide consideration, plan, and attempts.

**Results:**

A total of 10,463 adolescents, 14.8% of adolescents faced cyberbullying a past year. There was a higher prevalence of cyberbullying in youths aged 15-17 years (25 vs 26 vs 23%), which included more females to males (68 vs 32%).(p<0.0001) Caucasians (53%) had the highest number of responses to being cyberbullied compared to Hispanics (24%), African Americans (11%).(p<0.0001) There was an increased prevalence of cyberbullied youths with feelings of sadness/hopelessness (59.6 vs 25.8%), higher numbers considering suicide (40.4 vs 13.2%), suicide plan (33.2 vs 10.8%), and multiple suicidal attempts in comparison to non-cyberbullied.(p<0.0001) On regression analysis, cyberbullied adolescence had a 155% higher chance of feeling sad and hopeless [aOR=2.55; 95%CI=2.39-2.72], considered suicide [1.52 (1.39-1.66)], and suicide plan [1.24 (1.13-1.36)].

**Figure:**
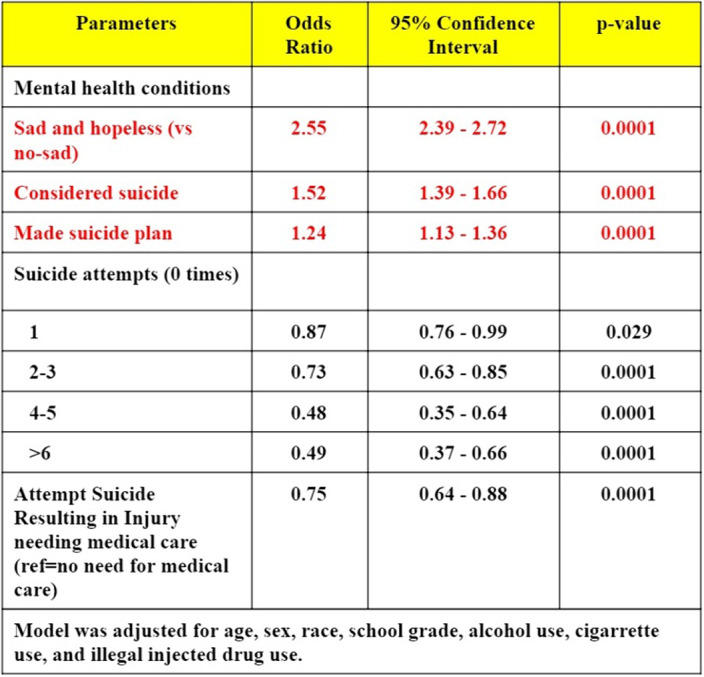

**Conclusions:**

In our study, cyberbullying was associated with negative mental health outcomes. Further research is warranted to examine the impact and outcomes of cyberbullying amongst adolescents and guiding the policies to mitigate the consequences.

**Disclosure:**

No significant relationships.

